# Current Blood Eosinophilia Does Not Predict the Presence of Pulmonary Hypertension in Patients with End-Stage Lung Disease

**DOI:** 10.3390/jcm14041120

**Published:** 2025-02-09

**Authors:** Michaela Barnikel, Nikolaus Kneidinger, Michael Gerckens, Carlo Mümmler, Alexandra Lenoir, Pontus Mertsch, Tobias Veit, Gabriela Leuschner, Andrea Waelde, Claus Neurohr, Jürgen Behr, Katrin Milger

**Affiliations:** 1 Department of Medicine V, LMU University Hospital, LMU Munich, Comprehensive Pneumology Center (CPC-M), Member of the German Center for Lung Research (DZL), 81377 Munich, Germany; michaela.barnikel@med.uni-muenchen.de (M.B.); nikolaus.kneidinger@medunigraz.at (N.K.); michael.gerckens@med.uni-muenchen.de (M.G.); carlo.muemmler@med.uni-muenchen.de (C.M.); alexandra.lenoir@med.uni-muenchen.de (A.L.); pontus.mertsch@med.uni-muenchen.de (P.M.); tobias.veit@med.uni-muenchen.de (T.V.); gabriela.leuschner@med.uni-muenchen.de (G.L.); andrea.waelde@med.uni-muenchen.de (A.W.); juergen.behr@med.uni-muenchen.de (J.B.); 2 Division of Pulmonology, Department of Internal Medicine, Lung Research Cluster, Medical University of Graz, 8036 Graz, Austria; 3 Institute of Lung Health and Immunity (LHI), Comprehensive Pneumology Center (CPC), Helmholtz Munich, Member of the German Center of Lung Research (DZL), 81377 Munich, Germany; 4 Department of Pulmonology and Respiratory Medicine, Robert-Bosch-Hospital, 70376 Stuttgart, Germany; claus.neurohr@rbk.de

**Keywords:** eosinophils, end-stage lung disease, pulmonary hypertension, hemodynamics

## Abstract

**Objectives:** To investigate the role of blood eosinophils in predicting PH in end-stage lung disease. **Methods:** We conducted a retrospective study of adults with CF, COPD, and ILD who underwent RHC during lung transplant evaluations (2010–2022). Patients were classified by the 2022 ECS/ERS PH guidelines with pulmonary function and laboratory tests, including hemograms. The eosinophil threshold was set at 0.30 G/L. **Results**: We analyzed 663 patients (n = 89 CF, n = 294 COPD, and n = 280 ILD). Severe PH was more common in ILD (16%) than in CF (4%) and COPD (7%) (*p* = 0.0002), with higher eosinophil levels in ILD (*p* = 0.0002). No significant correlation was found between eosinophil levels and hemodynamic parameters (PAPm, PVR, and CI) across CF, COPD, and ILD (PAPm: *p* = 0.3974, *p* = 0.4400 and *p* = 0.2757, respectively; PVR: *p* = 0.6966, *p* = 0.1489 and *p* = 0.1630, respectively; CI: *p* = 0.9474, *p* = 0.5705 and *p* = 0.5945, respectively), nor was a correlation observed in patients not receiving OCS. Linear regression analysis confirmed the lack of association (PAPm: *p* = 0.3355, *p* = 0.8552 and *p* = 0.4146, respectively; PVR: *p* = 0.6924, *p* = 0.8935 and *p* = 0.5459, respectively; CI: *p* = 0.4260, *p* = 0.9289 and *p* = 0.5364, respectively), controlling for 6-MWD, Nt-proBNP, and ICS/OCS dosages. ROC analysis indicated eosinophils were ineffective in distinguishing PH severity levels across these diseases (AUC 0.54, 0.51, and 0.53, respectively). The analysis of eosinophil levels measured 18 ± 6 months prior to baseline found no predictive correlation with the presence of PH either. Eosinophil levels did not differ significantly among PH groups, but eosinophilic COPD was linked to more unclassified PH, higher CO, and greater lung volumes than non-eosinophilic COPD. **Conclusions:** In our cohort of end-stage CF, COPD, and ILD patients, blood eosinophilia did not predict the presence of PH but was associated with hemodynamic parameters and lung volumes in COPD.

## 1. Introduction

Eosinophils are multifunctional leukocytes involved in tissue homeostasis, immune regulation, and inflammation [1]. Beyond their well-established role in defending against helminths and parasites, eosinophils have emerged as pivotal biomarkers in the diagnosis, phenotyping, and prognostication of asthma and chronic obstructive pulmonary disease (COPD). They also play a significant role in various other conditions, including atopic dermatitis, eosinophilic pneumonia, and eosinophilic granulomatosis with polyangiitis, as well as hypereosinophilic syndrome. Cardiovascular complications arising from eosinophilic-driven diseases, including endomyocardial fibrosis, myocarditis, endocarditis, and coronary arteritis, are major contributors to both morbidity and mortality [2,3,4]. These complications can severely impact patient outcomes, highlighting the extensive impact of eosinophilic activity. Additionally, eosinophils are linked to broader cardiovascular conditions, such as coronary artery calcification [5], although their role in chronic diseases remains unclear.

Even less is known about the impact of blood eosinophils in the development of pulmonary hypertension (PH). Experimental models suggest that eosinophils may significantly contribute to the pathogenesis of PH through mechanisms such as vascular inflammation inducing vascular remodeling and the development of arterial hypertrophy, which can lead to PH [6,7,8]. So far, there is evidence that eosinophilic COPD is associated with higher PAPm, increased PVR, and a greater likelihood of PH [9]. On the other hand, eosinophilic PAH may be linked to less severe hemodynamic impairment [10]. However, despite these findings, the inflammatory status of individuals has not yet been integrated into the clinical management of patients with pulmonary arterial hypertension (PAH) and PH. This gap in clinical practice is largely due to the scarcity of human studies on this topic.

To address this gap, our study evaluated the association of blood eosinophilia and PH in patients with end-stage lung diseases such as cystic fibrosis (CF), COPD, and interstitial lung disease (ILD). We aimed to clarify whether blood eosinophilia could serve as a reliable biomarker for predicting the presence of PH in these conditions by examining their association with functional and hemodynamic parameters.

## 2. Materials and Methods

### 2.1. Study Design

This retrospective, single-center study at the Department of Medicine V, University of Munich was approved by the central institutional ethics committee (#18-064).

### 2.2. Inclusion Criteria

We conducted a retrospective review of all adult patients diagnosed with CF, COPD, or ILD who underwent at least one right heart catheterization (RHC) at our center between January 2010 and December 2022 in a pre-transplant setting. RHC was performed as part of the evaluation and listing process for lung transplantation, regardless of the presence of clinical or echocardiographic signs of PH. Ventilation/perfusion (V/Q)-scan ruled out chronic thromboembolic pulmonary hypertension (CTEPH) in all patients.

### 2.3. Exclusion Criteria

Patients with the following diagnosis were excluded from our study: combined pulmonary fibrosis and emphysema (CPFE), sarcoidosis, systemic scleroderma, lymphangioleiomyomatosis, bronchiectasis, acute respiratory distress syndrome (ARDS), bronchiolitis obliterans organizing pneumonia (BOOP), eosinophilic lung diseases, e.g., comorbid asthma, and pre-re-transplant settings. Patients with incomplete hemodynamic data or hemograms, leading to insufficient classification, were also excluded, as well as those with a pulmonary capillary wedge pressure (PCWP) greater than 15 mmHg. Additionally, patients who underwent RHC while on specific vasoactive therapy or receiving high-dose systemic steroids (>30 mg per day) for exacerbation of underlying lung disease were excluded. Other immunosuppressive therapies, including biologics with a rheumatological indication, were not used as exclusion criteria.

### 2.4. Procedures

We conducted RHC by using a Swan–Ganz catheter and measured cardiac output (CO) by thermodilution. Patients were grouped according to the 2022 ECS/ERS guidelines for the diagnosis and treatment of PH [11]:

(1) No PH (mean pulmonary arterial pressure (PAPm) ≤ 20 mmHg)
(2) Unclassified PH (PAPm > 20 mmHg and pulmonal vascular resistance (PVR) ≤ 2 WU)
(3) Non-severe PH (PAPm > 20 mmHg and PVR > 2 WU and ≤5 WU)
(4) Severe PH (PAPm > 20 mmHg and PVR > 5 WU)

When multiple RHCs were performed, we included only the first available in our study. Baseline was defined as the time point of the RHC. Baseline characteristics included age, sex, and body mass index (BMI). Data on pulmonary function tests (PFTs), 6-min walk distance (6-MWD), capillary blood gas analysis, laboratory tests (including N-terminal pro brain natriuretic peptide (Nt-proBNP) and hemograms), as well as medication with oral corticosteroids (OCS) and inhaled corticosteroids (ICS), were collected contemporaneously within ±3 months of the RHC. Additionally, hemograms were obtained 18 ± 6 months prior to baseline, reflecting an earlier stage of the underlying lung disease. The eosinophil threshold was defined as 0.30 G/L. All medical examinations were conducted in accordance with applicable guidelines.

### 2.5. Statistics

Metric variables are presented as medians with 1st and 3rd quartile. To identify differences in continuous variables, Mann–Whitney U tests and Kruskal–Wallis tests were used for unpaired variables, and Fisher’s exact test was used for categorical variables. Post hoc tests were performed by using Dunn’s multiple comparison test. Wilcoxon tests were used to assess differences between paired variables. The correlations were examined using Spearman’s correlation coefficient and the analysis was conducted for the whole cohort as well as exclusively for patients not taking OCS. We performed multiple linear regression analysis and reported the results as *p*-values, while controlling for OCS and ICS dosages, among other factors. Receiver-operating characteristic (ROC) curves were used to evaluate the performance of a binary classification system by plotting the trade-offs between sensitivity and specificity with the area under the curve (AUC) indicating the model’s overall ability to distinguish between classes. *p*-values less than 0.05 were considered indicative of statistical significance. All statistical analyses were performed with GraphPad Prism version 8.3.0.

## 3. Results

### 3.1. Study Cohort and Baseline Characteristics

**Figure 1:**
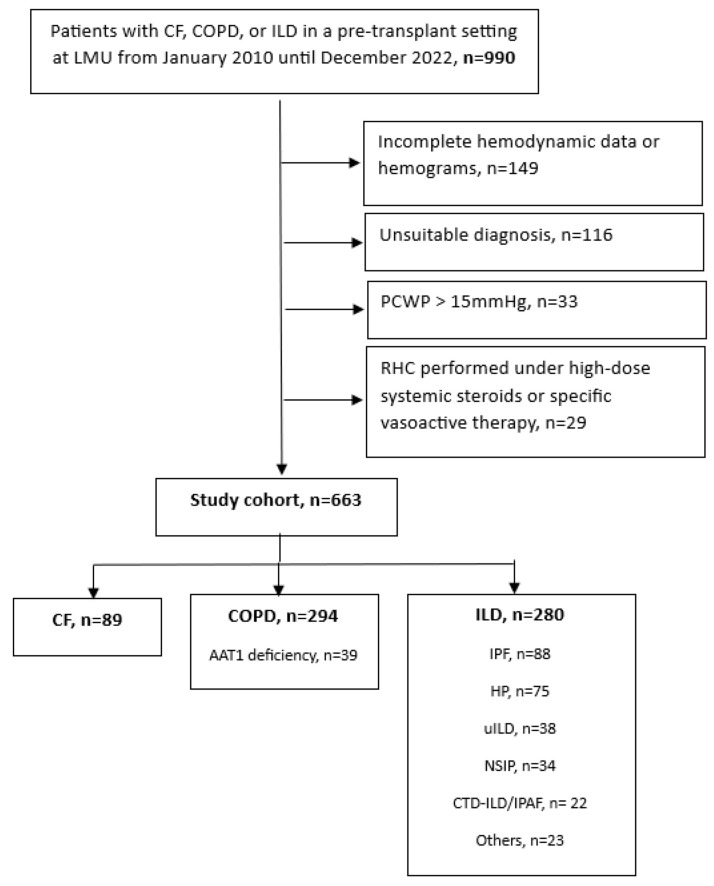
Study cohort.

During the study period, we reviewed 990 patients diagnosed with CF, COPD, or ILD as part of the lung transplantation evaluation process at our center. After applying the exclusion criteria, 327 patients were excluded: 149 due to incomplete data (n = 60 for incomplete hemodynamic data and n = 89 for incomplete hemograms), 116 due to an unsuitable diagnosis, 33 due to postcapillary PH, and 29 due to high-dose systemic steroids or specific vasoactive therapy. This left a final cohort of 663 patients: 89 patients with CF, 294 patients with COPD, and 280 patients with ILD. A detailed overview of the study cohort is shown in Figure 1.

Baseline characteristics varied as anticipated based on the underlying lung disease. In terms of PH classification, approximately half of our CF cohort met the criteria for non-severe PH (n = 50 patients, 56%), while severe PH was observed in only a few cases (n = 3 patients, 4%). In COPD, the proportion of patients with non-severe PH was numerically lower, while a higher percentage of patients had no PH at all compared to CF (COPD: n = 115 patients (39%); CF: n = 18 patients (20%), *p* = 0.0021). Severe PH in COPD was observed in 21 patients (7%). Notably, severe PH was more prevalent in patients with ILD compared to CF and COPD (ILD: n = 44 patients (16%); CF: n = 3 patients (4%), COPD: n = 21 patients (7%), *p* = 0.0002). Patients with ILD also had higher blood eosinophil levels compared to those with COPD and CF (ILD: 0.16 (0.07; 0.32) G/L, CF: 0.10 (0.01; 0.20) G/L; COPD: 0.13 (0.06; 0.14) G/L, *p* = 0.0004), despite a higher proportion of patients taking OCS. Additional baseline characteristics are presented in Table 1.

### 3.2. Predictive Value of Current Blood Eosinophilia for Pulmonary Hypertension in End-Stage Lung Disease

At time of RHC, eosinophil levels showed no significant correlation with hemodynamic parameters across CF, COPD, and ILD, including PAPm (CF: r = 0.091, *p* = 0.40; COPD: r = −0.0454, *p* = 0.44; ILD: r = 0.077, *p* = 0.28), PVR (CF: r = 0.042, *p* = 0.70; COPD: r = −0.085, *p* = 0.15; ILD: r = 0.099, *p* = 0.16), or cardiac index (CI) (CF: r = 0.011, *p* = 0.95; COPD: r = 0.033, *p* = 0.57; ILD: r = 0.033, *p* = 0.59). Due to the influence of OCS on blood eosinophil levels, correlation analyses were conducted exclusively in patients not on OCS. Yet, there was no significant correlation observed here as well: PAPm (CF: r = 0.010, *p* = 0.97; COPD: r = −0.052, *p* = 0.44; ILD: r = −0.104, *p* = 0.30), PVR (CF: r = −0.010, *p* = 0.97; COPD: r = −0.122, *p* = 0.08; ILD: r = −0.033, *p* = 0.78), or CI (CF: r = −0.065, *p* = 0.60; COPD: r = 0.099, *p* = 0.199; ILD: r = 0.05, *p* = 0.62).

Similarly, linear regression analysis found no significant association between eosinophil levels and PAPm (CF: 95%CI −13.98 to 4.890, *p* = 0.3355; COPD: 95%CI −1.630 to 1.963, *p* = 0.8552; ILD: 95%CI −8.042 to 3.327, *p* = 0.4146, respectively), PVR (CF: 95%CI −1.614 to 1.083, *p* = 0.6924; COPD: 95%CI −0.4287 to 0.3742, *p* = 0.8935; ILD: 95%CI −1.481 to 0.7852, *p* = 0.5459, respectively) or CI (CF: 95%CI −1.055 to 2.445, *p* = 0.4260; COPD: 95%CI −0.1907 to 0.2088, *p* = 0.9289; ILD: 95%CI −0.2839 to 0.5441, *p* = 0.5364, respectively) while controlling for 6-MWD, Nt-proBNP, ICS (beclometason equivalent) and OCS (prednisolone equivalent) dosages.

**Figure 2:**
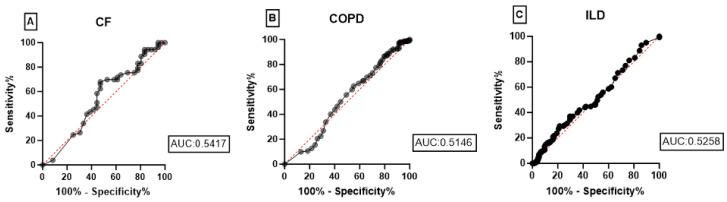
ROC curves of (**A**) CF, (**B**) COPD and (**C**) ILD.

ROC analysis indicated that current eosinophils were ineffective in distinguishing between non-severe or severe PH and no PH or unclassified PH in these end-stage lung diseases. The AUC for predicting the presence of non-severe or severe PH versus no PH or unclassified PH was 0.54 (95% CI: 0.4153 to 0.6680) in CF, 0.51 (95% CI: 0.4483 to 0.5809) in COPD, and 0.53 (95% CI: 0.4578 to 0.5938) in ILD (Figure 2). Using an eosinophil threshold of 0.30 G/L, the sensitivity was 0.83 and the specificity 0.19 in the CF cohort. For COPD, the sensitivity reached 0.87 with a specificity of 0.18, whereas in the ILD cohort, the sensitivity was 0.29 and the specificity 0.76. Using an eosinophil threshold of 0.15 G/L, the sensitivity was 0.70 and the specificity 0.47 for CF. For COPD, the corresponding values for sensitivity and specificity were 0.63 and 0.45, respectively, and for the ILD cohort, they were 0.50 and 0.49.

**Figure 3:**
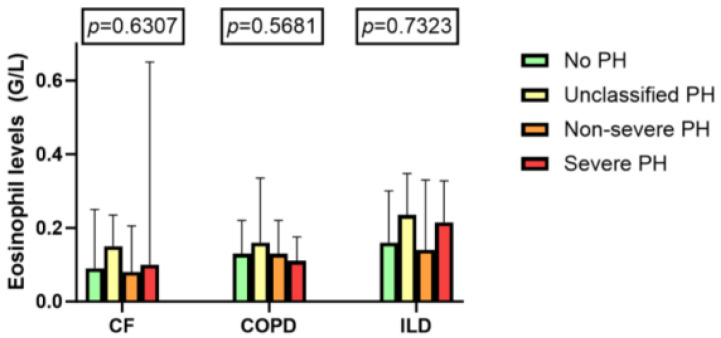
Blood eosinophil levels by PH classification; median with interquartile range.

Our analysis revealed no significant differences in current blood eosinophil levels among the various PH groups (CF: *p* = 0.63; COPD: *p* = 0.57; ILD: *p* = 0.73), Figure 3. However, ILD exhibited the highest eosinophil levels among the groups of PH.

### 3.3. Predictive Value of Prior Blood Eosinophilia for Pulmonary Hypertension in End-Stage Lung Disease

Next, we analyzed eosinophil levels measured 18 ± 6 months prior to baseline, reflecting an earlier stage of the underlying lung disease. Corresponding data were available for 37 out of 89 patients with CF, 118 out of 294 patients with COPD, and 89 out of 280 patients with ILD.

No significant differences in eosinophil levels over time were observed across CF, COPD, and ILD (CF: baseline 0.10 (0.01; 0.20) vs. 18 ± 6 months prior: 0.20 (0.10; 0.20); ∆ −0.02 (−0.1; 0), *p* = 0.0830; COPD: baseline 0.13 (0.06; 0.14) vs. 18 ± 6 months prior: 0.13 (0.06; 0.25); ∆ 0 (−0.06; 0.09), *p* = 0.8225; ILD: baseline 0.16 (0.07; 0.32) vs. 18 ± 6 months prior: 0.16 (0.08; 0.28); ∆ 0.01 (−0.03; 0.12), *p* = 0.0830). Furthermore, there were no differences in the changes in blood eosinophils between the cohorts (CF: ∆ −0.02 (−0.1; 0); COPD: ∆ 0 (−0.06; 0.09); ILD: ∆ 0.01 (−0.03; 0.12), *p* = 0.4011). Additionally, the difference in blood eosinophil levels between prior and baseline measurements (∆ eosinophils) did not vary significantly across the severity levels of PH (no PH, unclassified PH, non-severe PH, and severe PH), with corresponding *p*-values of 0.69, 0.79, and 0.26 for CF, COPD, and ILD, respectively.

In none of the three disease entities did changes in eosinophil levels (∆ eosinophils) correlate with the parameters defining and categorizing the severity of PH, such as PAP (CF: r = 0.052, *p* = 0.76; COPD: r = −0.059, *p* = 0.52; ILD: r = −0.051, *p* = 0.62) or PVR (CF: r = 0.162, *p* = 0.3; COPD: r = −0.03911, *p* = 0.67; ILD: r = −0.02486, *p* = 0.81). The same result was observed in the respective cohorts without OCS use, where no significant associations with PAP (CF: r = −0.04191, *p* = 0.85; COPD: r = −0.09233, *p* = 0.43; ILD: r = −0.08471, *p* = 0.69) or PVR (CF: r = 0.07821, *p* = 0.72; COPD: r = 0.0009959, *p* = 0.99; ILD: r = −0.03263, *p* = 0.85) were found.

### 3.4. Distinctive Aspects of Eosinophilic COPD

By categorizing patients according to their current blood eosinophil levels at baseline, using a threshold of 0.30 G/L, we observed that 19% of CF cases (n = 17 patients), 15% of COPD cases (n = 45 patients), and 28% of ILD cases (n = 79 patients) were eosinophilic (*p* = 0.0007). The classification of PH remained consistent across eosinophilic and non-eosinophilic groups in all underlying diseases. No significant differences were observed in clinical, functional, or hemodynamic parameters between eosinophilic and non-eosinophilic cohorts in CF and ILD (Tables S1 and S2). However, in COPD, significant differences emerged: unclassified PH was more frequent in eosinophilic COPD (*p* = 0.0054), with increased CO and CI (*p* = 0.0030 and *p* = 0.0420, respectively), but lower partial pressure of oxygen (pO_2_) levels (*p* = 0.0262). Additionally, total lung capacity (TLC) and residual volume (RV) were higher in eosinophilic COPD (*p* = 0.0106 and *p* = 0.0249, respectively) (Table 2).

## 4. Discussion

In our cohort of 663 patients with end-stage lung diseases, including CF, COPD, and ILD, the majority exhibited either no PH or non-severe PH. Severe PH was rare, occurring more frequently in patients with ILD, who also had higher blood eosinophil levels. However, current eosinophil levels did not correlate with the presence of PH across various disease groups, irrespective of the effects of OCS and ICS, as there were no significant differences in eosinophil levels among the different severity groups of PH. Similarly, previous eosinophil levels obtained 18 months prior to RHC, thus reflecting an earlier stage of the underlying lung disease, did not reveal any predictive association with pulmonary hypertension. Using a threshold of 0.30 G/L to classify eosinophilic status, we identified eosinophilic disease in 15% to 28% of cases. While no significant differences in clinical, functional, or hemodynamic parameters were observed between the non-eosinophilic and eosinophilic groups in the CF and ILD cohorts, some distinctions emerged in the COPD cohort. Specifically, patients with eosinophilic COPD had higher CO and CI but lower arterial pO_2_. Additionally, TLC and RV were elevated in the eosinophilic COPD group.

In our cohort of patients with end-stage lung diseases, neither current nor previous blood eosinophil counts were a valid biomarker for predicting the presence of PH across various disease entities, as there was no association between blood eosinophil levels and hemodynamic or functional parameters. This contrasts with findings from a recent study involving 106 COPD patients, where a similar percentage of eosinophilic COPD cases (using the same 0.30 G/L threshold) was identified. In that cohort, eosinophilic COPD was linked to higher mean PAPm, increased PVR, and a greater likelihood of PH, while total TLC was lower in this group. Notably, the eosinophilic group included significantly more patients with Global Initiative for Chronic Obstructive Lung Disease (GOLD) stage 2 COPD, and approximately 10% of the non-eosinophilic group exhibited a positive bronchodilator response [9]. However, in a cohort of patients with PAH, eosinophil levels exceeding 0.10 G/L appeared to be associated with less severe hemodynamic impairment, as indicated by a lower PAPm, reduced PVR, and higher central venous oxygen saturation (SvO_2_) [10]. We were unable to replicate these findings in our cohort, possibly due to differing eosinophil cut-off values and, more importantly, the fact that our study included patients with group 3 PH rather than those with PAH.

The role of eosinophils in the development of PH remains poorly understood and constitutes an important, yet largely unresolved, area of research. Current knowledge is primarily based on experimental models, which suggest that eosinophils contribute to the pathogenesis of PH through mechanisms such as vascular inflammation, remodeling, and arterial hypertrophy [6,7,8]. However, there is limited evidence regarding the potential clinical relevance of eosinophils in PAH [10]. So far, there is no relevant information for group 2 PH (PH associated with left heart disease). Regarding the significance of eosinophils in group 3 PH (PH associated with lung disease), including the cohort addressed in our study, only a few comparable studies exist [9], which, as previously discussed, have produced divergent results. For group 4 PH (CTEPH), we identified one study investigating ferroptosis-related potential biomarkers and immune cell characteristics [12]. Ferroptosis, a form of programmed cell death induced by iron-dependent lipid peroxidation, is increasingly recognized as an important mechanism in various disease processes, including CTEPH. This study found that immune infiltration analysis revealed a lower infiltration of eosinophils and neutrophils in CTEPH samples compared to controls, suggesting that both eosinophils and neutrophils may play a role in the pathological regulation of CTEPH [12].

These findings highlight the intricate role of eosinophils in PH and emphasize the necessity for further research to clarify their specific contributions across the different forms of pulmonary hypertension. While our study offers a modest contribution to this field, it reinforces the need for more comprehensive and targeted investigations to deepen our understanding.

Moreover, the comparability of our findings with previous studies is limited by cohort heterogeneity, including differences in diagnoses, disease stages, and thresholds used to define eosinophilic groups. Furthermore, the differing study cohorts exhibit diversity in terms of comorbidities and medication use, both of which may potentially influence eosinophil levels. Moreover, the thresholds for defining pulmonary hypertension have also changed in recent years, which impacts the prevalence of the condition and therefore affects the comparability of study results. It is also important to note that our study did not examine eosinophil levels in compartments beyond the peripheral blood, such as in sputum or bronchoalveolar lavage fluid—areas that may warrant further investigation in future research.

The observed association of increased eosinophil counts and more severe PH in the ILD group, despite lacking statistical significance or predictive value, warrants further exploration. The elevated eosinophil counts observed in ILD patients, despite higher doses of OCS, may reflect a more prominent role of eosinophils in the pathophysiology of ILD compared to conditions such as COPD or CF, potentially due to distinct inflammatory pathways or tissue-specific interactions. While it is conceivable that increased eosinophil levels could contribute to both tissue and vascular remodeling, thereby promoting the progression of both ILD and associated PH, this hypothesis remains speculative. The exact mechanisms underlying the role of eosinophils in the progression of ILD and PH have yet to be fully elucidated. It should also be considered that the absence of statistical significance might result from methodological factors such as a limited sample size or population heterogeneity, potentially obscuring a genuine but subtle association. Alternatively, the effect may only manifest in specific ILD subtypes, such as those with predominant eosinophilic inflammation. Although the current findings lack predictive value, they may point to a subgroup of ILD patients with distinct inflammatory profiles. Eosinophilic lung disease was excluded from the study cohort; however, the previously discussed effect of subtypes with predominant eosinophilic inflammation cannot be entirely excluded. To further address this point, additional studies focusing on precise subtype classification and their comparison would be of great interest. To further explore the lack of a predictive value of current blood eosinophil levels for pulmonary hypertension, despite higher eosinophil counts and more severe PH in ILD, we also analyzed eosinophil levels measured 18 ± 6 months prior to baseline, reflecting an earlier stage of the underlying lung disease. However, even after analyzing different time-points, which capture the dynamic course of this marker, no predictive association with pulmonary hypertension was found.

In our cohort of patients with eosinophilic COPD, unclassified PH was notably more prevalent. Given that these patients often exhibit elevated pulmonary blood flow [11], it is consistent to observe increased CO and CI within the eosinophilic COPD group. However, despite these higher CO levels, lower pO_2_ was also noted, indicating significant shunt volume and ineffective oxygenation. Additionally, we observed elevated TLC and RV, suggesting a greater degree of hyperinflation in eosinophilic COPD. This increased hyperinflation and subsequent alveolar expansion likely compress alveolar vessels, reducing the surface area available for gas exchange and causing a shift toward larger vessels, a phenomenon observed in COPD patients with PH [13].

Considering our study results, which showed a significantly higher TLC and RV in the eosinophilic COPD group, the necessity arises to discuss the potential influence of eosinophils on parenchymal processes. While the precise role of eosinophils in driving emphysema remains unclear, previous research has uncovered correlations between elevated blood eosinophil counts and signs of connective tissue damage. Notably, Doyle et al. investigated eosinophils and alveolar damage using a mouse model of chronic type 2 pulmonary inflammation, demonstrating that eosinophil-derived interleukin (IL)-13 stimulates alveolar macrophages to produce MMP-12, a key mediator of emphysema [14]. These findings suggest a potential role for eosinophils in lung damage, independent of conventional disease markers or smoking-related factors. Earlier studies further support this, showing that IL-13 overexpression leads to a phenotype characterized by significant emphysema and lung enlargement [15]. Moreover, the SPRIOMICS study revealed significantly higher emphysema indices on CT scans in patients with sputum eosinophils ≥1.25% compared to those with lower sputum eosinophil levels. This association was not observed with blood eosinophils at a threshold of 0.20 G/L, as blood eosinophil counts did not reliably predict sputum eosinophil levels in this cohort [16]. This study suggests that eosinophil-driven mechanisms may contribute to the development of emphysema, potentially explaining the observed increases in TLC and RV and their subsequent impact on alveolar vessels in eosinophilic COPD within our study cohort. However, this observation was not reproduced in our obstructive and eosinophilic CF cohort, indicating that additional factors characteristic of COPD might be involved. The influence of smoking is also critical, as it is known to cause the loss of alveolar capillaries [17,18], potentially resulting in an increased shunt volume, which was observed in our cohort. Dysregulated angiogenesis may play a significant role in COPD, with pulmonary vascular remodeling potentially involving a dynamic process characterized by elevated vascular endothelial growth factor (VEGF) expression in the early stages and reduced levels in later stages [19].

In the discussion of the potential influence of inflammatory mediators on the development of PH in lung disease, the investigation of therapeutically targeted interleukins is of significant interest. Biologic therapies that modulate IL-5 and IL-13 signaling pathways are well-established in clinical practice due to their profound effects on eosinophils, and consequently, on eosinophil-driven diseases. This is particularly well established in the treatment of severe asthma. However, the role of therapeutic IL-5 or IL-13 blockade in the development of PH remains unclear. Clinically, these are largely distinct patient cohorts: severe asthma patients undergoing biologic therapy and those with PH associated with lung disease, with the former seldom being evaluated for PH, and in most cases, not invasively. Other interleukin blockade therapies, typically used in the context of rheumatologic diseases, may offer greater potential in this regard. Nevertheless, these considerations remain theoretical and require further validation through clinical studies. Taking all these considerations into account further strengthens the evidence that the factors contributing to the development of pulmonary hypertension in lung diseases are multifactorial. Eosinophils may represent a potential piece of this puzzle, potentially influencing both vascular and parenchymal processes.

When discussing PH in lung diseases, it is essential to consider the complex pathomechanisms involved, including the potentially unique role of eosinophils. Known contributors to PH in lung diseases such as COPD and CF include chronic hypoxemia [20,21] and pulmonary vascular remodeling, which is driven by inflammation and mediated by immune cells [7,22,23]. The significance of low DLCO as a key parameter in predicting high mortality in both idiopathic pulmonary arterial hypertension (IPAH) and PH associated with interstitial lung disease highlights the relevance of these mechanisms [24]. Recent findings on lung phenotypes in IPAH, a primarily vascular-driven disease, provide new insights into the relationship between PH and lung diseases [25]. The disconnect observed in ILD between the severity of PH and the extent of underlying lung disease, as seen in lung function tests or fibrotic findings on HRCT [26], as well as the inverse association between the severity of PH and emphysema in end-stage COPD [27], suggests the presence of significant drivers beyond parenchymal involvement.

In summary, the situation is highly complex, with many of the pathomechanisms underlying PH in lung diseases remaining unclear. Additionally, it is still uncertain whether eosinophils have both a direct effect on PH and an indirect influence, potentially through their impact on airway disease and emphysema in COPD, thus influencing spirometric and body plethysmographic values. Given these complexities, future classifications may need to delineate distinct phenotypes based on the underlying lung disease, its severity, and the predominant pathomechanism driving PH.

Our study is limited by selection bias, its exclusive focus on end-stage lung diseases in a pre-transplant context, and the inherent heterogeneity of the study cohort. Particularly, the exclusive focus on end-stage lung diseases may have led to the exclusion of potential associations between eosinophils and the presence of PH at an earlier stage of the underlying lung disease, which occurred more than two years prior, thereby precluding any conclusions regarding this in the present study. Our study examined whether current blood eosinophilia is linked to the presence of PH, with both parameters measured simultaneously. Longitudinal data were available only for eosinophil levels, while a longitudinal assessment of the hemodynamic profile was not conducted. Therefore, we cannot make any conclusions about the development of PH over time. Furthermore, due to the observational character of our study, residual confounding cannot be excluded. In addition, because of the cross-sectional design, no assumptions about causality can be made. Our study is subject to the limitations inherent in a retrospective design, such as the risk of incomplete data, confounding factors, and selection bias. Also, we can only describe associations, but no causality. To gain a clearer understanding of the role of eosinophils in vascular processes, further research in real-life cohorts diagnosed with PAH are warranted. Additionally, it is crucial to consider the wider array of factors that might affect eosinophil levels in these analyses.

## 5. Conclusions

In conclusion, we could not establish a predictive role of either current or previous eosinophil counts in peripheral blood for PH in a heterogenous group of patients, including those with end-stage CF, COPD, and ILD. However, there is evidence indicating that eosinophil levels correlate with lung volumes in COPD. Given the high complexity of the pathomechanisms contributing to PH in lung disease, along with the multifaceted roles of eosinophils, further research is crucial.

## Figures and Tables

**Table 1:** Baseline characteristics grouped according to the underlying disease.

	CF	COPD	ILD	*p*-Value
Patients, n	89	294	280	
Male, n (%)	47 (53)	149 (51)	179 (64)	**0.0042** ^c^
Age (years)	29 (26; 37)	58 (53; 62)	58 (51; 62)	**<0.0001** ^a,b^
BMI (kg/m^2^)	19 (17; 20)	22 (19; 25)	25 (22; 28)	**<0.0001** ^a,b,c^
PH classification				
No PH	18 (20)	115 (39)	108 (38)	**0.0021** ^a,b^
Unclassified PH	18 (20)	37 (13)	22 (8)	**0.0062** ^b^
Non-severe PH	50 (56)	121 (41)	106 (38)	0.09
Severe PH	3 (4)	21 (7)	44 (16)	**0.0002** ^b,c^
PFT				
FVC (L)	1.8 (1.4; 2.4)	1.8 (1.4; 2.3)	1.7 (1.2; 2.1)	**0.0334** ^c^
FVC (%)	44 (36; 50)	48 (40; 61)	42 (34; 53)	**<0.0001** ^a,c^
TLC (L)	5.8 (5.0; 7.1)	8.1 (6.9; 9.3)	3.2 (2.6; 4.0)	**<0.0001** ^a,b,c^
TLC (%)	104 (92; 116)	136 (121; 151)	52 (44; 63)	**<0.0001** ^a,b,c^
RV (L)	3.9 (3.3; 5.2)	6.1 (5.0; 7.2)	1.5 (1.2; 1.9)	**<0.0001** ^a,b,c^
RV (%)	260 (210; 321)	290 (246; 342)	71 (54; 87)	**<0.0001** ^b,c^
FEV1 (L)	0.9 (0.7; 1.1)	0.6 (0.5; 0.8)	1.4 (1.1; 1.9)	**<0.0001** ^a,b,c^
FEV1 (%)	25 (22; 30)	22 (17; 27)	47 (37; 57)	**<0.0001** ^a,b,c^
Tiffenau (%)	50 (40; 60)	36 (31; 41)	90 (84; 95)	**<0.0001** ^a,b,c^
RHC				
mPAP (mmHg)	25 (21; 30)	22 (19; 26)	23 (18; 29)	**0.0007** ^a,b^
mRAP (mmHg)	5 (3; 7)	6 (4; 8)	3 (2; 5)	**<0.0001** ^a,b,c^
PCWP (mmHg)	9 (6; 12)	9 (7; 11)	6 (4; 10)	**<0.0001** ^b,c^
PVR (WU)	2.5 (1.9; 3.2)	2.4 (1.8; 3.2)	2.8 (2.0; 3.9)	**<0.0001** ^b,c^
CO (L/min)	6.3 (5.6; 7.5)	5.4 (4.7; 6.0)	5.6 (4.7; 6.4)	**<0.0001** ^a,b^
CI (L/min/m^2^)	4.0 (3.4; 4.6)	3.1 (2.7; 3.4)	3.0 (2.7; 3.4)	**<0.0001** ^a,b^
SvO_2_ (%)	67 (64; 73)	72 (67; 74)	71 (68; 75)	**0.0001** ^a,b^
6MWD (m)	375 (290; 450)	245 (175; 315)	300 (200; 388)	**<0.0001** ^a,b,c^
NT-proBNP (pg/mL)	90 (45; 205)	73 (41; 136)	94 (49; 211)	**0.0067** ^c^
pO_2_ (mmHg) *	65 (60; 75)	63 (55; 71)	60 (53; 66)	**<0.0001** ^b^
pO_2_ (mmHg) ^#^	63 (58; 71)	56 (48; 62)	56 (51; 62)	**<0.0001** ^a,b^
pCO_2_ (mmHg)	43 (39; 49)	44 (39; 49)	41 (38; 45)	**0.0001** ^b,c^
Eosinophils (G/L)	0.10 (0.01; 0.20)	0.13 (0.06; 0.14)	0.16 (0.07; 0.32)	**0.0004** ^b,c^
ICS, n (%)	42 (47)	176 (60)	30 (11)	**<0.0001** ^a,b,c^
ICS dose (beclometason equivalent, µg/d)	1000 (400; 1900)	800 (400; 1000)	450 (225; 760)	**0.0002** ^b,c^
OCS, n (%)	20 (22)	64 (22)	180 (64)	**<0.0001** ^b,c^
OCS dose (prednisolone equivalent, mg/d)	7.5 (5; 10)	6.25 (5; 10)	7.5 (5; 10)	0.24

Parameters are given as median with 1st and 3rd quartile; * all, ^#^ no oxygen supply; ^a^ significant: CF vs. COPD; ^b^ significant: CF vs. ILD; ^c^ significant: COPD vs. ILD. Bold data indicate significance.

**Table 2:** Non-eosinophilic vs. eosinophilic COPD.

	Eosinophils < 0.3 G/L	Eosinophils ≥ 0.3 G/L	*p*-Value
Patients, n	249	45	
Male, n (%)	124 (50)	26 (58)	0.34
Age (years)	58 (53; 62)	56 (53; 59)	0.08
BMI (kg/m^2^)	22 (19; 25)	22 (19; 26)	0.74
PH classification			
No PH	99 (40)	16 (36)	0.62
Unclassified PH	25 (10)	12 (27)	**0.005**
No-severe PH	107 (43)	14 (31)	0.14
Severe PH	18 (7)	3 (6)	0.99
PFT			
FVC (L)	1.8 (1.4; 2.3)	1.8 (1.5; 2.3)	0.78
FVC (%)	48 (40; 60)	49 (38; 58)	0.39
TLC (L)	7.8 (6.8; 9.3)	8.9 (7.9; 9.6)	**0.01**
TLC (%)	135 (121; 151)	143 (118; 165)	0.12
RV (L)	5.8 (4.9; 7.2)	6.7 (5.3; 7.9)	**0.025**
RV (%)	287 (243; 342)	318 (269; 376)	**0.033**
FEV1 (L)	0.6 (0.5; 0.8)	0.6 (0.5; 0.8)	0.45
FEV1 (%)	21 (17; 27)	22 (16; 25)	0.29
Tiffenau (%)	35 (30; 41)	35 (30; 38)	0.45
RHC			
mPAP (mmHg)	22 (19; 26)	23 (19; 26)	0.70
mRAP (mmHg)	6 (4; 8)	6 (4; 7)	0.74
PCWP (mmHg)	9 (7; 12)	9 (8; 12)	0.35
PVR (WU)	2.4 (1.9; 3.2)	2.1 (1.5; 3.2)	0.1399
CO (L/min)	5.3 (4.7; 6.0)	6.0 (5.2; 6.6)	**0.003**
CI (L/min/m^2^)	3.0 (2.6; 3.4)	3.2 (2.8; 3.8)	**0.042**
SvO_2_ (%)	71 (67; 75)	72 (69; 77)	0.18
6MWD (m)	245 (180; 310)	245 (166; 325)	0.84
NT-proBNP (pg/mL)	75 (43; 140)	65 (33; 91)	0.13
pO_2_ (mmHg) *	63 (55; 71)	61 (51; 71)	0.33
pO_2_ (mmHg) ^#^	56 (48; 63)	51 (45; 58)	**0.026**
pCO_2_ (mmHg)	43 (39; 49)	45 (40; 50)	0.21
Eosinophils (G/L)	0.11 (0.05; 0.17)	0.38 (0.34; 0.60)	**0.0001**

Parameters are given as median with 1st and 3rd quartile; * all, ^#^ no oxygen supply. Bold data indicate significance.

## Data Availability

The data presented in this study are available on request from the corresponding author. The data are not publicly available due to restrictions concerning privacy.

## Supplementary Materials

The following supporting information can be downloaded at: https://www.mdpi.com/article/10.3390/jcm14041120/s1, Table S1: Non-eosinophilic vs. eosinophilic CF; Table S2: Non-eosinophilic vs. eosinophilic ILD.
