# Supplementary material for: Current Blood Eosinophilia Does Not Predict the Presence of Pulmonary Hypertension in Patients with End-Stage Lung Disease
Source: J Clin Med. 2025 Feb 9;14(4):1120. doi: 10.3390/jcm14041120 (PMC11856528; doi:10.3390/jcm14041120)
Supplement: Supplementary file 1 [file jcm-14-01120-s001.zip › jcm-3308909-supplementary.pdf]

Table S1. Non-eosinophilic vs eosinophilic CF.

	**Eosinophils < 0.3 G/L**	**Eosinophils ≥ 0.3G/L**	**p-value**
Patients, n	72 (81)	17 (19)	
Male, n (%)	38 (53)	9 (53)	0.6060
Age (years)	29 (25;37)	29 (25;37)	0.9959
BMI (kg/m^2)	19 (17;20)	18 (17;19)	0.3967
PH classification<br>No PH<br>Unclassified PH<br>No-severe PH<br>Severe PH	 14 (19)<br>14 (19)<br>42 (59)<br>2 (3)	 4 (23)<br>4 (23)<br>8 (48)<br>1 (6)	 0.7408<br>0.7408<br>0.4274<br>0.4748
PFT<br>FVC (l)<br>FVC (%)<br>TLC (l)<br>TLC (%)<br>RV (l)<br>RV (%)<br>FEV1 (l)<br>FEV1 (%)<br>Tiffenau (%)	 1.8 (1.4;2.4)<br>44 (33;52)<br>6.0 (4.9;7.3)<br>104 (91;117)<br>3.9 (3.2;5.4)<br>264 (209;329)<br>0.9 (0.7;1.1)<br>24 (21;30)<br>49 (40;61)	 1.7 (1.5;2.3)<br>43 (38;50)<br>5.6 (5.2;6.6)<br>106 (93;119)<br>3.9 (3.3;4.5)<br>256 (222;317)<br>1.0 (0.7;1.3)<br>27 (23;31)<br>53 (44;63)	 0.7033<br>0.7756<br>0.6908<br>0.9575<br>0.6069<br>0.8830<br>0.3070<br>0.3396<br>0.3938
RHC<br>mPAP (mmHg)<br>mRAP (mmHg)<br>PCWP (mmHg)<br>PVR (WU)<br>CO (l/min)<br>CI (l/min/m^2)<br>SvO_2 (%)	 26 (21;30)<br>5 (3;7)<br>9 (6;12)<br>2.5 (1.9;3.2)<br>6.3 (5.7;7.5)<br>4.1 (3.4;4.6)<br>67 (64;73)	 25 (20;31)<br>5 (2;7)<br>8 (7;11)<br>2.7 (1.8;3.8)<br>6.0 (5.3;7.5)<br>3.8 (3.2;4.5)<br>68 (65;71)	 0.9773<br>0.4219<br>0.9524<br>0.5992<br>0.5413<br>0.3350<br>0.9400
6MWD (m)	360 (283;465)	383 (299;428)	0.8902
NT-proBNP (pg/ml)	91 (43;207)	84 (40;198)	0.6269
pO_2 (mmHg)*<br>pO_2 (mmHg)#<br>pCO_2 (mmHg)	64 (60;75)<br>63 (57;72)<br>43 (39;52)	67 (60;75)<br>66 (59;70)<br>42 (39;48)	0.5622<br>0.7644<br>0.5880
Eosinophils (G/L)	0.05 (0.02;0.14)	0.42 (0.33;0.61)	**0.0001**

Parameters are given as median with 1st and 3rd quartile; *all, #no oxygen supply

Table S2. Non-eosinophilic vs eosinophilic ILD.

	**Eosinophils < 0.3 G/L**	**Eosinophils ≥ 0.3G/L**	**p-value**
Patients, n	201	79	
Male, n (%)	124 (62)	56 (71)	0.1671
Age (years)	58 (52,62)	58 (47;63)	0.2496
BMI (kg/m^2)	25 (22;28)	25 (22;28)	0.9683
PH classification<br>No PH<br>Unclassified PH<br>No-severe PH<br>Severe PH	 80 (40)<br>15 (8)<br>75 (37)<br>31 (15)	 28 (36)<br>7 (9)<br>31 (39)<br>13 (16)	 0.5856<br>0.8053<br>0.7854<br>0.8560
PFT<br>FVC (l)<br>FVC (%)<br>TLC (l)<br>TLC (%)<br>RV (l)<br>RV (%)<br>FEV1 (l)<br>FEV1 (%)<br>Tiffenau (%)	 1.6 (1.2;2.1)<br>41 (34;51)<br>3.2 (2.5;3.9)<br>51 (44;63)<br>1.5 (1.1;1.8)<br>70 (54;88)<br>1.4 (1.1.;1.8)<br>47 (37;57)<br>90 (83;95)	 1.8 (1.3;2.3)<br>44 (34;58)<br>3.4 (2.7;4.2)<br>53 (45,63)<br>1.5 (1.2;1.9)<br>72 (53;86)<br>1.6 (1.2;1.9)<br>50 (38;60)<br>91 (84;96)	 0.1117<br>0.4734<br>0.2140<br>0.5684<br>0.4124<br>0.8988<br>0.0960<br>0.4098<br>0.4492
RHC<br>mPAP (mmHg)<br>mRAP (mmHg)<br>PCWP (mmHg)<br>PVR (WU)<br>CO (l/min)<br>CI (l/min/m^2)<br>SvO_2 (%)	 23 (18;28)<br>3 (2;5)<br>6 (4;9)<br>2.7 (2.1;4.1)<br>5.6 (4.6;6.3)<br>3.0 (2.6;3.4)<br>72 (68;75)	 24 (19;31)<br>3 (2;5)<br>6 (4;10)<br>3.1 (2.0;4.6)<br>5.8 (4.8; 6.5)<br>3.1 (2.6;3.5)<br>71 (66;74)	 0.4095<br>0.3524<br>0.9997<br>0.4183<br>0.0923<br>0.1969<br>0.0561
6MWD (m)	300 (188;390)	300 (213;388)	0.7722
NT-proBNP (pg/ml)	94 (48;223)	95 (54;177)	0.8552
pO_2 (mmHg)*<br>pO_2 (mmHg)#<br>pCO_2 (mmHg)	60 (53;65)<br>55 (50;62)<br>41 (38;46)	61 (54;68)<br>59 (53;66)<br>41 (38;45)	0.2481<br>0.0916<br>0.9067
Eosinophils (G/L)	0.1 (0.05;0.19)	0.42 (0.34;0.58)	**0.0001**

Parameters are given as median with 1st and 3rd quartile; *all, #no oxygen supply
